# Is Reference a Relation of Equivalence or Sameness?

**DOI:** 10.1007/s40614-025-00473-1

**Published:** 2025-09-11

**Authors:** Elberto A. Plazas

**Affiliations:** Fundación Universitaria Konrad Lorenz, Bogotá, Colombia

**Keywords:** Reference, Meaning, Stimulus equivalence, Relational frame theory, Speaker

## Abstract

The stimulus equivalence (SE) paradigm has become a central explanatory framework for language and complex symbolic behavior within behavior analysis. Its explanatory power rests on three core assumptions: (1) human symbolic behavior is grounded in the semantic relation between words and their referents; (2) this relation is one of equivalence; and in consequence (3) there is a transfer of stimulus functions between words and their referents. These assumptions are also endorsed by relational frame theory (RFT), although considering equivalence as a consequence of a relation of sameness within a relational frame of coordination. However, this article shows that the referential relation is neither reflexive, symmetrical, nor transitive, and therefore cannot be characterized as one of equivalence or sameness, invalidating (2) and (3). It is also shown that other attempts to support (2) or (3), based on the Fields-Place principle or contextual control, fail to achieve their aim. It is argued that between the behavior of the speaker and the listener, there is a functional asymmetry that grounds the asymmetry of the referential relation, and typical SE and RFT experimental paradigms cannot capture it. Finally, some consequences for the study of the SE phenomenon and the study of symbolic behavior from the perspective of behavior analysis are discussed.

Meaning in general, and linguistic meaning in particular, is a subject of interest to many disciplines, including philosophy of language, semiotics, linguistics, information science, cognitive science, and behavioral science. However, the term "meaning" primarily functions as an everyday language term rather than a technical concept, rendering its definition elusive (e.g., Ogden & Richards, 1923; Smith, 1997). Nonetheless, in ordinary communication, we often manage to understand one another when discussing the meaning of a linguistic expression, interpreting someone’s intent behind an utterance, or recognizing when an expression appears meaningless. Considering the fundamental role of linguistic exchanges in shaping human social and cultural life, investigating how linguistic expressions convey meaning constitutes a critical scientific endeavor in understanding human behavior.

The relationship between behavior analysis (BA) and the problem of linguistic meaning has been, at best, ambiguous. Pre-Skinnerian behaviorism provided Pavlovian interpretations of the connotative and denotative meanings of words as conditioned stimuli (e.g., Staats, 1968, ch. 2). It should be noted that Osgood's theory of meaning, grounded in Hull's mediational theory of behavior, was once regarded as a leading behaviorist approach to linguistic meaning (e.g., Osgood, 1953, ch. 16; Osgood, 1956). By contrast, Skinner’s *Verbal Behavior* (1957) rejected attempts to explain linguistic meaning in conventional terms, arguing that such efforts rely on a traditional framework that bases meaning on the speaker's ideas. Furthermore, Skinner contended that defining the meaning of a linguistic expression inevitably involves referencing other linguistic expressions, which themselves require explanation. Instead, Skinner proposed identifying meaning with the independent variables that influence behavior:

Technically, meanings are to be found among the independent variables in a functional account, rather than as properties of the dependent variable. When someone says that he can see the meaning of a response, he means that he can infer some of the variables of which the response is usually a function. (Skinner, 1957, p. 14)

This perspective places meaning within the broader context of behavior in general, whether verbal or nonverbal. However, it has little direct relevance to the specific question of how statements have meaning in communicative exchanges. The Skinnerian framework exhibits an eliminative stance toward traditional concepts of *communication*, *meaning*, and *reference*, focusing instead on the contingencies that shape speaker and listener behavior. (Although, as we will see later, the Skinnerian view of verbal episodes implicitly addresses referencing.)

By contrast, the stimulus equivalence (SE) paradigm emerged in the 1980s as a framework within BA to address the nature and functioning of linguistic meaning, particularly the reference, and its influence on symbolic behavior (Sidman, 1994; Sidman & Tailby, 1982). Over the past 4 decades, SE paradigm has become a cornerstone of BA, providing the foundation for the relational frame theory (RFT; Hayes, 1991a, 1991b; Hayes, Barnes-Holmes et al., 2001). RFT extends SE paradigm to other relations, such as differentiation, opposition, and comparison. As the basis for contextual behavioral science (Zettle et al., 2016), RFT has gained significant prominence in recent years. A central feature of RFT is its adherence to the semantic principles underlying the SE paradigm.

This article aims to analyze whether the SE paradigm offers an adequate explanation of reference or the semantic relation between words and their referents. But to begin, we must first present the SE paradigm.

## What Is the Stimulus Equivalence Paradigm?

First of all, the SE paradigm is a scientific paradigm in its own right. Like the Pavlovian or operant conditioning paradigms, it consists of a particular experimental preparation, in which subjects or participants are exposed to special training conditions and changes in their behavior are obtained that are predictable and replicable to other subjects or participants. This procedure is taken as an explanatory model, which is extended to certain phenomena of daily life outside the laboratory in order to understand its functioning.

The SE paradigm originated from an applied study conducted by Sidman with a young man with intellectual disabilities. The study demonstrated the indirect acquisition of a basic reading comprehension repertoire (Sidman, 1971). The participant was trained to form two types of arbitrary conditional relations: dictated words to pictures and dictated words to written words. Once the participant achieved proficiency in these relations, emergent performances—responses not explicitly trained—spontaneously appeared. For example, the participant accurately matched pictures to written words and vice versa without direct instruction. These emergent behaviors illustrated a remarkable efficiency in learning. In particular, training 40 relations (20 dictated word → picture and 20 dictated word → written word) resulted in the emergence of 40 novel relations (20 picture → written word and 20 written word → picture). It is important to note that the relationships between trained and emergent stimuli were arbitrary, ruling out stimulus generalization as an explanation. Sidman’s findings were soon replicated in studies involving other participants with intellectual disabilities (Sidman & Cresson, 1973; Sidman et al., 1974).

By the early 1980 s, Sidman formalized the SE paradigm in its present form (Sidman & Tailby, 1982; Sidman et al., 1982). This paradigm involves training arbitrary conditional relations between stimuli and subsequently observing emergent behaviors that exhibit the mathematical properties of equivalence: reflexivity, symmetry, and transitivity. For instance, in a typical two-choice matching-to-sample procedure, a participant trained to relate sample stimulus A1 with comparison B1 and A2 with B2, and later B1 with C1 and B2 with C2, might demonstrate reflexivity if he or she matches a stimulus with itself (e.g., A1 with A1). The participant display symmetry if reverses the trained relations (e.g., selecting A1 when B1 is presented as the sample). And he or she display transitivity if relates stimuli indirectly connected through a common stimulus (e.g., choosing C1 when A1 is the sample, based on prior training of A1–B1 and B1–C1). Finally, the participant is said to display equivalence if he or she demonstrates symmetrical transitivity (e.g., selecting A1 when C1 is the sample).

One remarkable aspect of the SE paradigm is its efficiency. For example, training four relations (A1–B1, A2–B2, B1–C1, B2–C2) leads to the emergence of 14 new relations, including 6 of reflexivity (A1–A1, A2–A2, B1–B1, B2–B2, C1–C1, C2–C2), 4 of symmetry (B1–A1, B2–A2, C1–B1, C2–B2) and 4 of transitivity and equivalence (A1–C1, A2–C2, C1–A1, C2–A2). These emergent relations form equivalence classes (e.g., A1, B2, and C1 on the one hand, and A2, B2, and C2 on the other) in which stimuli become interchangeable concerning their behavioral functions. This functional equivalence is evidenced by the transfer of stimulus functions, where a function trained to one class member (e.g., A1) automatically transfers to others (e.g., B1 and C1) without direct training (Sidman, 1986; Dougher & Markham, 1994). Transfer of different stimulus functions has been observed between members of equivalence classes, such as high or low rates of responding (Barnes & Keenan, 1993; Catania et al., 1989a, 1989b), conditioned reinforcer or punisher functions (Hayes et al., 1987; Hayes et al., 1991), contextual control (Dougher et al., 2002; Kohlenberg et al., 1991; Gatch & Osborne, 1989; Lynch & Green, 1991), sequence ordering (Lazar, 1977; Wulfert & Hayes, 1988), self-discriminative responding (Dymong & Barnes, 1994), and even respondent elicitation and extinction (Augustson & Dougher, 1997; Dougher et al., 1994; Roche & Barnes, 1997). Nevertheless, the formation of stimulus classes and the transfer of function may be affected by the similarity of the functions of the stimuli (Tyndall et al., 2004).

Interest in the transfer of functions between formally dissimilar stimuli did not begin with Sidman’s paradigm, but had previously been investigated within the topic of mediated generalization or transfer, through the paired-associate paradigm (e.g. Peters, 1935; Cofer & Foley, 1942; Jenkins; 1963, Palermo, 1962. For a recent review of this field, see Eilifsen & Anrtzen, 2021). However, Sidman rejected the mediational behaviorism (Hull, 1939) that dominated this type of research, in which emergent performances were the effect of a covert mediating response (Sidman et al., 1974). Instead, Sidman proposed that equivalence classes emerge directly from the reinforcement contingencies of training (Sidman, 1977). He later incorporated the mathematical concept of equivalence relations to rigorously define and test their properties (Sidman & Tailby, 1982). Although some researchers have debated whether the mathematical definition adequately characterizes the phenomenon (e.g., Dougher & Markham, 1996; Pilgrim, 2016; Pilgrim & Galizio, 1996; Saunders & Green, 1992; Zentall et al., 2008), Sidman’s approach remains the most influential.

The SE paradigm has demonstrated strong reliability in humans, including adults (e.g., Lazar, 1977) and typically developing children (Lazar et al., 1984; Sidman & Tailby, 1982; Sidman et al., 1986). However, emergent equivalence relations are generally absent in verbally unskilled children (Devany et al., 1986) and nonhuman animals (D’Amato et al., 1985; Dugdale & Lowe, 2000; Lipkens et al., 1988; Sidman et al., 1982). These findings suggested a link between the emergence of equivalence relations and linguistic capabilities (e.g., Hayes, 1989; Hayes et al., 1987). Longitudinal studies indicate that infants develop emergent relational abilities only after acquiring early language skills (Lipkens et al., 1993; Luciano et al., 2007). In nonhuman animals, properties such as symmetry and transitivity have been observed, but only after extensive explicit training in some or all of the properties that define equivalence (e.g., Schusterman & Kastak, 1993, 1998; Frank & Wasserman, 2005; Urcuioli, 2008); and spontaneous emergence akin to humans remains elusive (for recent reviews of the topic, see Galizio & Bruce, 2018; Lionello-DeNolf, 2021). Equivalence relations remain a relevant phenomenon in the debate on the continuity of human behavior with respect to nonhuman behavior (e.g., Dymond, 2014; Hughes & Barnes-Holmes, 2014; McIlvane, 2014).

In applied contexts, the SE paradigm has been widely adopted due to its learning efficiency, giving rise to equivalence-based instruction (EBI). This instructional approach has been successfully employed to teach diverse skills across populations, including individuals with developmental disabilities, autism, and learning difficulties (e.g., Blair & Dorsey, 2021; De Rose et al., 1996; Lynch & Cuvo, 1995; Saunders et al., 2003; Stromer & Mackay, 1992). Applications include skills such as mand behavior (e.g., Murphy & Barnes-Holmes, 2009), reading (e.g., De Rose et al., 1996), second language vocabulary acquisition (e.g., Haegele et al., 2011), academic concepts in college students (Fienup et al., 2011), statistical concepts (e.g., Fields et al., 2009; Fienup & Critchfield, 2011), single-subject design (Lovett et al., 2011; Walker & Rehfeldt, 2012), the value of money (Keintz et al., 2011), musical skills (e.g., Hayes, et al., 1989; Griffith et al., 2018), nutritional information of food items (Arntzen & Eilertsen, 2020), among others.

Furthermore, the SE paradigm underpins RFT, which extends its principles to a broader range of relational responses, such as opposition, comparison, hierarchy, etc.; aiming to provide a comprehensive explanation of human cognition (Hayes, Barnes-Holmes et al., 2001). A relational frame is defined as arbitrarily applicable higher-order relational responding that adheres to the properties of mutual entailment, combinatorial entailment, and transformation of stimulus functions (Hayes, 1991a, 1991b, 1994). Within RFT, relational frames are conceptualized as higher-order operant behaviors that develop from an individual's learning history. In the standard experimental design of RFT, relational responding is brought under the control of contextual stimuli, followed by training these responses using arbitrary stimuli; in order to then evaluate mutual entailment, combinatorial entailment, and the transformation of stimulus functions (e.g., Steele & Hayes, 1991). In RFT, equivalence relations are regarded as outcomes of the coordination frame, which involves applying a sameness relation under contextual control to stimuli that are formally dissimilar (Barnes-Holmes et al., 2004; Hayes, 1991a, 1991b). In particular, it is proposed that the coordination frame originates from a learned history of bidirectional relational responding, a process that typically develops in humans during language acquisition (Hayes, Fox et al., 2001a, 2001b). As a result, symmetrical mutual entailment is considered the core property of coordination frames in RFT. It should be noted, however, experimental paradigms often involve training contextual control of the sameness relation using stimuli that are formally similar, rather than dissimilar (e.g., Steele & Hayes, 1991).

## The SE Paradigm as an Explanation of Symbolic Behavior

The stimulus equivalence (SE) paradigm exhibits remarkable behavioral properties, including the emergence of novel conditional discriminations, learning efficiency, and functional equivalence among dissimilar stimuli. Sidman further proposed that SE underpins the semantic relationship between words and their referents. In his words:

Stimulus classes formed by network of equivalence relations establish a basis for referential meaning. The equivalence paradigm provides exactly the test that is needed to determine whether or not a particular conditional discrimination involves semantic relations. (Sidman & Tailby, 1982, p. 20, italics mine)

And also,

The assumption one is making here is that the observed conditional relations (e.g., if [the word is] red, then [select] the red hue; if green, then green, etc.) are also equivalence relations; that the words are equivalent to their “referents.” (Sidman et al., 1986, p. 2)

The proposal is that between words and their referents a relation of equivalence is established, and the meaning of words is reduced to this relation:"... whenever people do use these terms [meaning, symbolism, and reference], we will find (by appropriate tests) that the words and their referents will be related by equivalence"(Sidman, 1994, p. 562). Because of these equivalence relations, words acquire their symbolic nature:"In the simplest case, a word does become equivalent to ‘the thing it stands for.’ That is why people call words ‘symbols’” (Letter to Day, October 10, 1986, cited in Sidman, 1994, p. 562). Two central assumptions may be identified from this proposal:

- S1: The semantic relations between words and their referents constitute the foundation of human symbolic behavior.
- S2: The semantic relations between words and their referents consist of equivalence relations.

As can be seen, S1 and S2 do not imply each other; each can be true independently of the other. S1 is broader and likely to be accepted across different theoretical perspectives. On the other hand, S2 is more contentious, but it constitutes the core of the proposal. If both assumptions hold, equivalence relations could serve as the explanatory basis for human symbolic behavior.

Sidman supported S2 apparently for two main reasons. First, word–referent relations are typically arbitrary, much like the arbitrary stimulus relations established in the SE paradigm. This arbitrariness aligns with the essence of the symbolic because, in animal cognition research, symbolic matching is equated with arbitrary matching (e.g., Carter & Werner, 1978; Sidman, 1994, pp. 128, 190; Zentall, 1998). Given that, in the SE paradigm, equivalence relations emerge between stimuli that are arbitrarily related, and words and their referents relate arbitrarily, we could infer S2. And assuming S1, we could also infer that equivalence is the core or the symbolic. Second, if words and referents share an equivalence relation, they belong to an equivalence class, enabling functional transfer between them (Sidman, 1992, 1994). For example, a learned response to a word can transfer to its referent and vice versa. This functional transfer can explain phenomena such as rule-governed behavior and the treatment of symbols as their referents. For instance, if one learns that the word"dog"represents dogs and subsequently has a negative experience with a dog, the word"dog"might elicit an avoidance response. On the other hand, if one hears the statement"dogs are dangerous,"the avoidance response to the word “dangerous” may generalize to real dogs. Such observations illustrate the power of words to influence behavior without direct environmental contact, as described in rule-governed behavior (Catania et al., 1989a, 1989b, 1990). From this, a third assumption can be derived:

- S3: Functional transfer occurs between words and their referents, explaining rule-governed behavior and the tendency to respond to symbols as though they are their referents.

Table 1 shows the S1–S3 assumptions. S3 is a natural consequence of S2, given the observed SE phenomenon. As Sidman noted:

... we often react to words and other symbols as if they are the things or events they refer to. Even though we do not treat word and referent as equal in all respects, we attribute some of the same properties to both. This treatment of linguistic forms as equivalent to their referents permits us to listen and read with comprehension, to work out problems in their absence, to instruct others by means of speech or text, to plan ahead, to store information for use in the future, and to think abstractly—all of these by means of words that are spoken, written, or thought in the absence of the things and events they refer to. (Sidman, 1994, p. 3)

**Table 1:** Core Assumptions of the Semantic Theory of the SE Paradigm

Assumption	Statement
S1	The semantic relations between words and their referents constitute the foundation of human symbolic behavior.
S2	The semantic relations between words and their referents consist of equivalence relations.
S3	Between words and their referents, transfers of stimulus functions occur.

As a consequence, the SE paradigm has been proposed as the main basis for explaining complex human symbolic cognition, including reading comprehension (Sidman, 1990), categorization (Fields et al., 1991; Galizio et al., 2001), social categorization (Roche & Barnes, 1996; Watt et al., 1991), analogical reasoning (Stewart et al., 2004), rule-governed behavior (Sidman, 1994; Hayes et al., 1989, 1991), grammar (Lazar, 1977; Wulfert & Hayes, 1988), creativity, and induction (Sidman, 1994).

Likewise, RFT supports S1, S2, and S3. Regarding S2, Hayes stated that “[a] frame of coordination is one that is central to those types of verbal relations termed ‘referential.’” (Hayes, 1991a, 1991b, p. 29). As for S1, RFT posits that the coordination frame and the equivalence relations it entails form the foundation of relational responding, upon which other relational frames are constructed (see, e.g., Barnes-Holmes et al., 2013; Hughes & Barnes-Holmes, 2016a). Relational responding serves as the foundation for relational reasoning, which supports cognition and intelligence (McLaughlin et al., 2020).

This article critically evaluates S2, with implications for S3, including the conception of reference as a coordination frame in RFT. I will make a brief reference to S1 in the final section.

## Is the Reference Relation Reflexive, Symmetrical, and Transitive?

The phenomenon of linguistic meaning is highly complex. To narrow our focus and avoid dealing with all its facets, this discussion concentrates on the referential meaning of linguistic expressions. An additional justification for this approach is that referential meaning is the most relevant type for assessing S2. In linguistics and the philosophy of language, reference is typically understood as the relation between a linguistic expression and the extralinguistic entity it denotes or designates (e.g., Sainsbury, 2008; Sterelny, 1997; Sullivan, 2006; cf. L. Hayes, 1991a, 1991b). The referent of a linguistic expression is the object or state of affairs to which the expression directs our attention. For example, when a listener responds to the mand “Hand me the stapler” by picking up a stapler rather than a notebook, pencil, or other item, and the response is reinforced by the speaker, the word “stapler” can be said to have fulfilled its referential function.

For Sidman (1994, p. 2), reference is central to meaning and symbolism: “One kind of word meaning is symbolic reference: many words are symbols; they refer to other things or events.” According to his proposal, the reference relation entails an equivalence relation between the word and its referent, characterized by reflexivity, symmetry, and transitivity:

I venture to claim that whenever we talk about word-meaning-referent, an equivalence relation will be found to exist between the word and its referent. This is the selfsame equivalence relation that elementary mathematics defines quite elegantly and precisely in terms of reflexivity, symmetry, and transitivity. (Note: bidirectionality—symmetry—is not enough.) (p. 566)

If the reference relation is indeed an equivalence relation, then it must satisfy these three properties. This claim lies at the heart of S2. For example, if the relation between the word “dog” and the class of animals it denotes is one of reference, then such a relation would simultaneously be reflexive, symmetrical, and transitive. Let us examine this claim.

### Reflexivity

If the reference relation is reflexive, the word “dog” would refer to itself, and, likewise, an individual dog would refer to itself. However, these assumptions are implausible. Since at least the time of St. Augustine, signs have been understood as objects that remit to others than themselves (Augustine, 1995, § II.1; cf. Augustine, 1968, § VIII.24). Symbols, as a subset of signs (e.g., Deacon, 1997; Peirce, 1992–1998; Place, 1995/1996), would also adhere to this principle—except in rare cases where an object signifies itself iconically, as in perceptual recognition (see Plazas, 2023, § 6.4). This is particularly evident with words. If the word “dog” refers to the class of animals it denotes, it does not simultaneously refer to itself, as it is not a member of that class. The same reasoning applies to proper nouns, such as “Fido.” Although language can sometimes be self-referential, instances of this are rare and often contrived. For example, whereas syntactic terms such as “word” or “noun” may refer to themselves, the pronoun “I” does not. When I use “I,” I refer to the person writing this text, not the word itself. Therefore, reflexivity is not a property of reference relation; rather, the reference relation is typically antireflexive.

## Transitivity

Let me continue with transitivity and leave symmetry for last, because the latter has larger implications. If the reference relation were transitive, anything that refers to a word would also refer to the word’s referent. Sidman’s examples of equivalence classes often include relations between written and spoken words, translations between languages, synonyms, lower- and upper-case letters, and numerals and their corresponding quantities (e.g., Sidman & Tailby, 1982; Sidman, 1990, 1992, 1994). For instance, there is said to be an equivalence relation between the written word “dog,” the spoken word/dog/, and the animal. If the written word refers to the spoken word, and the spoken word refers to the animal, then the written word should also refer to the animal. Likewise, if “perro” in Spanish refers to “dog” in English, and “dog” refers to the animal, “perro” should also refer to the animal.

However, this reasoning is problematic. Consider the written word “dog” and its spoken counterpart/dog/. Both refer to the class of animals they denote, but the written word does not refer to the spoken word per se. For example, the sign “Beware of the dog” does not use “dog” to refer to the spoken word/dog/but to the animal behind the fence. Although there is functional equivalence between written and spoken words in their referential functions, this equivalence does not establish mutual referentiality. The same argument applies to translations, synonyms, number systems and their names, and other analogous relations.

In addition, there are counterexamples to transitivity. For instance, the expression “common noun” refers to the word “dog” as well as to other nouns like “cat” or “bird.” Whereas “dog” refers to my dog Fido, “common noun” does not, because my dog is not a common noun. Likewise, the expression “three-letter word” or “word beginning with d refers to ‘dog,’” but they refer neither to Fido nor to the class of dogs. Therefore, the reference relation cannot be considered transitive.

### Symmetry

A relation is symmetrical if it is bidirectional, so that the relation between A and B is identical to that between B and A. Examples of symmetrical relations include being a sibling, a spouse, or being adjacent. In contrast, asymmetrical relations include being a parent, being taller, or causing an event. In Sidman’s theory, symmetry is a key feature of equivalence relations, as he posits that such relations naturally arise from reinforcement contingencies. In particular, all elements positively associated within a reinforcement contingency are bidirectionally related (Sidman, 2000). Symmetry also plays a pivotal role in RFT. According to RFT, the coordination frame, which involves equivalence relations, is learned through the training of multiple exemplars of word → referent and referent → word bidirectional relations in the early acquisition of language. When these two repertories are coordinated, their unity becomes controlled by certain contextual cues, such as the phrases"is the same as"or"is like."Later, when these cues are applied to sets of stimuli that are not formally similar, the stimuli become related to each other through a bidirectional sameness relation (Hayes, Fox et al., 2001a, 2001b). Given the coordination frame's foundational role in the emergence of more complex relational frames, RFT suggests that bidirectional relational training during early language acquisition serves as a critical gateway for human subjects to relational framing of events (e.g., Hughes & Barnes-Holmes, 2016a, 2016b).

If the reference relation were symmetrical, it would imply that if A refers to B, then B also refers to A. But is this truly the case? For instance, if the name *Fido* refers to my dog, does my dog, in turn, refer to his name? This seems highly implausible and challenges our conventional understanding of the reference relation. Although the referent may exert control on the speaker's tact behavior, as Skinner (1957) suggested, this stimulus control is not a relation of reference. My dog does not refer to its name *Fido*. Consider another example: the noun phrase “the word dog” refers to *dog*, but *dog* does not refer to the noun phrase “the word dog.” Instead, *dog* refers to the class of four-legged animals that bark, wag their tails, and are commonly kept as pets. Certainly, my dog does not refer to the noun phrase “the word dog.” As a result, the symmetry required for what Sidman termed the equivalence relation does not hold in this case either. In everyday understanding, reference and meaning are seen as directional relations from words to things, not vice versa. To assume otherwise would contradict our intuitive and practical use of these concepts.

Goodman (1968) famously criticized the idea that meaning is grounded in similarity, as similarity is both reflexive and symmetrical, whereas the meaning relation is neither. Although Goodman’s argument specifically addressed similarity as a basis for meaning, it also applies to equivalence relations. It highlights that meaning is not typically understood as a bidirectional relation. As Horne and Lowe (1996) aptly stated, “the relation between a name and that which it names is fundamentally asymmetrical” (p. 234). Later in this discussion, I will provide further arguments supporting the fundamental asymmetry of the reference relation.

In conclusion, the reference relation is neither reflexive, symmetric, nor transitive. As a result, it cannot be considered an equivalence relation, which mean that S2 should be rejected. If this argument is sound, then it challenges the theoretical underpinnings of Sidman’s paradigm and, by extension, RFT. However, this is not a new argument, and it had already been recognized earlier by Place (1995/1996); but he proposed a way out of this problem. In the next section, I will evaluate Place's solution.

## The Fields-Place Principle and Its Limitations

Place (1995/1996) initially expressed skepticism toward the stimulus equivalence (SE) paradigm as an explanation for the meaning of symbols, noting that the meaning relation is neither reflexive, symmetrical, nor transitive. However, influenced by Fields, he later proposed a potential solution to this issue. According to Place, symbols denote objects not because they are in an equivalence relation with those objects, but because they are in an equivalence relation with a natural sign associated with those objects. He articulated this idea in what he termed"Fields's Principle,"though it might be more aptly named the"Fields–Place Principle."This principle is expressed as follows:

A symbol is a sign which designates its object, not by virtue of a naturally-occurring association between sign and significatum, but by virtue of its membership of a Sidman stimulus equivalence class among whose other members is at least one sign which does derive its semantic function from such a naturally-occurring association and transfers that function to other members of the class. (p. 10)

According to Place, every sign is a discriminative stimulus that directs attention toward its referent. A linguistic expression becomes a symbol insofar as it is an arbitrary, conventionally established sign that derives its discriminative value from its membership in an equivalence class that includes natural signs of the object. For instance, the proper name Margaret Thatcher denotes the person in question because the name belongs to an equivalence relation with natural signs such as her visual appearance or the sound of her voice. Place argued that relations of reflexivity, symmetry, and transitivity hold between the symbol and the natural signs of the object it denotes, making this a case of equivalence (Place, 1995/1996, p. 8).

However, Place’s proposal does not support S2, because by the Fields–Place Principle, the denotation or reference of a symbol to an object is not grounded in an equivalence relation between the symbol and the object itself. Instead, the equivalence relation pertains to the symbol and the natural signs of the object, which constitutes a departure from Sidman’s framework and RFT. Although the Fields–Place Principle is not inherently incompatible with S2, its underlying motivation is the rejection of S2.

Place’s proposal is an attempt to uphold the idea that, despite the falsity of S2, equivalence relations are nevertheless involved in symbolic behavior. However, several issues with this proposal can be identified. First, Place fails to explain how the name"Margaret Thatcher"comes to stand in an equivalence relation with natural signs of Margaret Thatcher. Place seems to assume that natural signs have a referential function concerning their objects—one that is subsequently transferred to the names with which they enter into an equivalence relation. Yet it is highly implausible that natural signs possess such a referential function. As will be argued later, there are strong reasons to believe that the referential relation is established through communicative exchanges between speakers and listeners, and it is uncommon for speakers to use the natural signs of their referents to denote them. Last, we learn about Mrs. Thatcher through various media, such as television images, photographs, and voice recordings. However, this is different from knowing someone's name through direct contact. In this case, is the name associated with the person or the signs representing that person? How can we distinguish between the person and their signs? If this distinction cannot be made, then the Fields–Place Principle cannot overcome S2.

## The Contextual Control Counterargument and Its Limitations

The previous sections have given sufficient reasons to reject S2. In § 2 it was stated that S3 is a consequence of S2. Recall that S3 postulates that there is a transfer of stimulus function between words and their referents. Now, although S2 is rejected, it remains logically possible to maintain S3 on independent grounds. However, S3 is not a universally accepted proposition, and several authors have pointed out counterexamples to it. Skinner (1957) famously observed,"We do not behave toward the word'fox'as we behave toward foxes..."(p. 87). Likewise, Horne and Lowe (1996) observed,"We do not sit on the printed word'chair'when we see it, nor can we sit on the spoken word'chair'"(p. 235). The typical response of proponents of the SE paradigm to these issues has been to appeal to contextual control (e.g., Hayes, 1991a, 1991b; Sidman, 1992, 1994).

Sidman (1992) acknowledged that certain stimulus functions of referents are nontransferable to words: “We do not try to eat the word ‘bread,’ or swat the word ‘fly’” (p. 22). However, he argued that this phenomenon could be explained through contextual control, whereby equivalence classes are modulated by contextual cues. For example, in a classic study by Bush et al. (1989), participants were trained in conditional relations (AB and BC) under two different auditory contexts (high tone and low tone), which resulted in the formation of different equivalence classes for each context. This demonstrated that contextual stimuli could control the configuration of equivalence classes—a concept Sidman referred to as second-order conditional discrimination or five-term contingencies (Sidman, 1986). Likewise, in RFT, contextual cues play a central role in determining the type of relational frame applied to stimuli and how properties such as mutual entailment, combinatorial entailment, and transformation of stimulus functions manifest (Hayes, 1991a, 1991b; Hayes, Fox et al., 2001a, 2001b; Hughes & Barnes-Holmes, 2016a).

In the context of word–referent relations, contextual control is indeed useful and even necessary for explaining lexical ambiguity, where the same word may have multiple meanings. Contextual cues—linguistic (e.g., phrases or sentences) or extralinguistic—help disambiguate meaning. For instance, in the sentence “The fisherman went to the bank,” the reference to “fisherman” clarifies the intended meaning of “bank.” Likewise, in “There’s a bat in the attic,” extralinguistic cues, such as preparing for a baseball game, can clarify the intended referent. Both the SE paradigm and RFT successfully account for such phenomena.

However, Sidman’s (1992, 1994) suggestion that contextual control might explain the limitations in function transfer between words and referents raises issues. Many stimulus functions are inherently tied to the physical properties of referents and cannot transfer to words (e.g., one can pet a dog but not the word “dog”). Likewise, many properties of words—such as their phonemic construction, utterability in speech acts, or ability to combine with other words—are not transferable to referents. Sidman’s proposal implies that contextual cues signal when transfer is possible, yet such limitations are permanent and do not vary across contexts. Thus, contextual control offers no explanatory value for these constraints.

Now, having provided sufficient reasons to reject S2, the defender of S3 bears the burden of proof to explain how functional equivalence between linguistic expressions and their referents could occur in such a way that function transfer is achieved without assuming an equivalence relation (or sameness, in RFT). However, many cases interpreted as instances of function transfer between words and their referents could be explained through more parsimonious means. To extend a previous quote from Skinner (1957):

In at least several alleged cases of function transfer from referents to linguistic expressions, it could be said that the latter evoke anticipatory responses regarding the former through behavioral processes that do not involve an equivalence relation. Likewise, many instances of what is considered rule-governed behavior could be explained without appealing to S3. But even if it is argued that But we do not behave toward the word “fox” as we behave toward foxes, except in a limited case. If we are afraid of foxes, the verbal stimulus fox, which we have heard in the presence of real foxes, will evoke an emotional reaction; if we are hunting, it may create the condition we call excitement or delight. (p. 87)

There are phenomena associated with the stimulus functions of words or with rule-governed behavior that do not seem to be explicable in terms of more basic behavioral principles, it would seem preferable to avoid any appeal to S3 unless there is some way to explain how it occurs, upon departing from S2. As a result, there appear to be more reasons to remain skeptical of S3 than to support it.

## The Inability of the SE Paradigm to Capture the Referential Asymmetry between Speaker and Listener Behavior

One of the main reasons for endorsing S2 within the SE paradigm lies in the implicit assumption that the act of referring can be reduced to responding correctly within a matching-to-sample (MTS) task. In the SE experimental paradigm, symmetry is said to occur when a subject consistently selects a stimulus B1 rather than another stimulus B2 conditionally on sample stimulus A1, and when A1 is selected over A2 conditionally on B1. In both cases, the response topography consists of selecting, touching, picking up, or otherwise choosing one stimulus from among a set of comparison stimuli. Thus, the exchange of positions between the sample stimulus and the S+ is taken as evidence of symmetry.

The SE paradigm assumes that something analogous to the experimental procedure takes place between words and their referents. In an MTS task, a word may be presented as the sample stimulus and several objects as comparison stimuli, including the object that is conventionally referred to by the word in the verbal community and that would function as the S+. The consistent selection of this object would be considered a correct response. In other types of MTS trials, the object that previously functioned as the S+ is now used as the sample stimulus, and a set of words—including the one that conventionally refers to the object—is presented as comparison stimuli. The consistent selection of the conventionally associated word would again be considered correct. In this way, the procedure demonstrates a symmetrical relation between a word and its referent within the MTS context.

Sidman (1994) believed that one of the methodological advantages of the SE paradigm is that it allows for a clear and objective assessment of language comprehension and production behaviors. Correct pairings of word → object would serve as evidence of language comprehension, whereas object → word pairings would be interpreted as instances of language production, akin to tacting behavior. These performances in comprehension and production would be constitutive of listener and speaker behavior, respectively. Moreover, each would assess one direction of the bidirectional referential relation between word and referent. This would support both the symmetry between speaker and listener behavior and the validity of S2.

However, the SE experimental paradigm aims to study the semantic referential relation abstracted from any communicative interaction, speech act, or verbal episode. Yet it is debatable whether the referential relation between words and referents can be sustained independently of such interactions. As Hayes acknowledged (Hayes & Grundt, 1997), reference is understood not only as a semantic relation between linguistic expressions and referents but also as an action. One might say that the referential relation is enacted through the act of referring. In John Searle’s (1969, ch. 4) theory of speech acts, reference is viewed as a common element across a wide variety of illocutionary acts, including assertions, questions, commands, promises, etc. Moreover, speaking of referential relations outside of acts in which such relations are instantiated seems to run counter to the pragmatist and functionalist approach of behavior analysis.

Despite this, the SE experimental paradigm does not involve communicative interactions in which referential acts are performed. In general, a participant is seated in front of a computer or similar device that presents the sample and comparison stimuli for each trial and delivers feedback—without involving any communicative exchange with the apparatus. The communicative interaction occurs earlier, when the participant receives instructions from the experimenter, and later when compensation for participation is given. The same applies to traditional experimental procedures used in RFT research (e.g., Steele & Hayes, 1991), as well as to other alternative procedures such as the relational evaluation procedure (e.g., Stewart et al., 2004), the IRAP (e.g., Hughes & Barnes-Holmes, 2013), and the function acquisition speed test (FAST; Watters et al., 2023).

However, an even more challenging issue for the SE paradigm is that the act of referring involves a functional asymmetry between speaker and listener behavior. According to Searle (1969, § 4.5), reference must fulfill an identifying function for the referent:

A necessary condition for the successful performance of a definite reference in the utterance of an expression is that either the expression must be an identifying description or the speaker must be able to produce an identifying description on demand. (p. 88)

Thus, the act of reference is performed by the speaker in order to direct the listener’s attention toward that which the speaker is referring to. As L. Hayes (1991a, 1991b) explains: “It is this orientation of the listener with respect to the things spoken of that is meant by the concept of reference, and it is only by way of verbal stimulation that it can come about with any degree of efficiency” (p. 12). Through a linguistic act, the speaker (typically) refers to something in the extralinguistic world, and the listener is thereby oriented to that part of the world so as to act upon it in accordance with what is"said"through the speaker’s linguistic act. For this reason, the referential relation is asymmetrical and follows the direction linguistic expression → referent, but not in the reverse direction, as demonstrated in § 3.

The functional asymmetry between speaker and listener in the act of referring is also present in Skinner’s (1957) description of the verbal episode. Although Skinner rejected the notion of reference as a semantic relation, his analysis of tact behavior implicitly contains the idea of reference as an act: “The listener’s response to a tact is obviously influenced by the correspondence between form of response and controlling stimulus” (p. 86). That controlling stimulus is the nonverbal stimulus that governs the speaker’s verbal (typically linguistic) tact response, and simultaneously serves as the referent for the listener:

In very general terms we may say that behavior in the form of the tact works for the benefit of the listener by extending his contact with the environment, and such behavior is set up in the verbal community for this reason. (p. 85)

This act of reference is also implicit in the mand. When a speaker says, “Pass me the salt,” the listener is expected to reach for the salt and not the pepper or some other object. The very notion of verbal behavior in Skinner’s framework seems to entail referentiality, because verbal behavior is defined as that which produces a change in the world not directly, but through the behavior of another individual—the listener. But how can such change be achieved with precision if not through a referential act? It is precisely because of this definition that Skinner did not consider listener behavior to be verbal. Listener behavior was understood by Skinner (1969, 1989) instead as rule-governed behavior, in which a rule is a “contingency-specifying stimuli.” This formulation of rules and rule-governed behavior has not been free of criticism (e.g., Hayes & Hayes, 1991a, 1991b); however, part of what seems to be conveyed by the expression “specifying contingencies” relates to this referential character that rules have in orienting the behavior of the listener.

Against the view that referential action is unidirectional and asymmetric, it might be argued that many instances exist in which the individual acting as a listener also functions as a speaker. This is clearly evident in conversations, which are part of many communicative exchanges. However, the fact that a listener also acts as a speaker does not seem necessary for a verbal or communicative episode to occur. In fact, what is definitional and distinctive about the roles of speaker and listener in a communicative episode is the difference in how each relates to the referential act. It is precisely for this reason that it makes sense to speak of turn-taking or role-switching in conversation.

One of the limitations of Skinner’s analysis is that listener behavior is not verbal (Hayes & Hayes, 1989). This has led to alternative definitions of verbal behavior. According to RFT (Hayes, Fox et al., 2001a, 2001b), verbal behavior consists of relationally framing events, in accordance with contextual control, to apply certain kinds of relational responding to specific stimuli, resulting in mutual entailment, combinatorial entailment, and transformation of stimulus functions. Within this framework, both speaker and listener behaviors are equally verbal. Although it is acknowledged that “verbal communication from the speaker’s point of view becomes verbally purposive. Verbal behavior is used to produce verbally known effects in a listener;” it is nonetheless affirmed that “[s]peaking with meaning and listening with understanding both involve arbitrarily applicable derived relational responding” (Barnes-Holmes et al., 2001, p. 116). Thus, the RFT notion of verbal behavior does not appear to be sensitive to the functional asymmetry between speaker and listener roles in referential acts.

In conclusion, the SE experimental paradigm creates the illusion that language production and comprehension performances—as well as speaker and listener behaviors—are symmetrical and stand in a relation of equivalence (or identity, in RFT), because it abstracts away from the referential acts involved in communicative interactions. However, referential acts reveal a functional asymmetry between speaker and listener behaviors (or listener roles), which is tied to the asymmetrical nature of the referential relation itself—something the SE paradigm is unable to capture.

## Some Concluding Words

In summary, the SE paradigm and RFT have grounded their explanatory capacity for human symbolic behavior on assumptions S1, S2, and S3, with S2 serving as the central premise (§ 2). This article has argued against S2. It has demonstrated that the referential relation between words and their referents is neither reflexive, symmetric, nor transitive, and thus does not constitute a relation of equivalence or sameness (§ 3). This issue cannot be resolved by appealing to the *Fields*–*Place Principle* (§ 4). In addition, it also does not appear feasible to uphold S3 independently based solely on an appeal to contextual control (§ 5). If a referential relationship exists between words and their referents, it is sustained by the speaker's acts of reference directed toward the listener during verbal interactions. This interaction is unidirectional and asymmetrical, where the speaker's act of reference connects the listener to the specific part of the extralinguistic world being referenced. The fact that many behavior analysts have upheld such an untenable theory of semantic relations seems due to their reliance on experimental paradigms that abstract away from communicative acts, operating under the assumption that these paradigms capture the referential relation without involving the act of referring (§ 6). Many of the arguments presented here address facts well-known to linguists, philosophers of language, semioticians, and cognitive scientists (e.g., Crane, 2015, § 2.3; Goodman, 1968; Sterelny, 1997; Sullivan, 2006). For these fields, a theory asserting that the referential relationship is one of equivalence or sameness would be regarded as no more than an eccentric curiosity.

The critique presented here is not intended to deny the existence of the SE phenomenon. On the contrary, substantial research demonstrates that humans with a certain level of linguistic development spontaneously form equivalence relations, as defined by Sidman's criteria. In my view, the phenomenon of SE appears to provide significant evidence of the discontinuity between nonhuman and human behavior. Likewise, the contributions of equivalence-based instruction in various applied contexts are undeniable. Therefore, this article is not a call to cease research within the paradigm, whether in basic or applied studies. However, it should be clear that neither the amount of evidence supporting the phenomenon nor its applied utility constitutes an argument in favor of S2 or S3. Instead, this is an invitation to stop investigating the paradigm under the assumption that it reveals something fundamental about word–referent semantic relations or human symbolic behavior in general.

I believe the arguments presented here have implications for the question of the origin of the SE phenomenon. If S2 is false, then the formation of SE relations is not a fundamental phenomenon underlying the acquisition and use of language. As a result, SE would be a complex phenomenon that itself requires explanation. Explanations based on naming behavior (e.g., Dugdale & Lowe, 1990; Horne & Lowe, 1996; Place, 1995. For a recent review of how naming theory and SE and RFT accounts are similar and different, see Regaço et al., 2025) or classification behavior (e.g., Plazas & Peña, 2016) have been proposed. However, it may be the case that multiple pathways lead to the emergence of equivalence relations (Plazas, 2024).

This article has presented critiques of S2 and S3, but S1 has been left unaddressed. However, the rejection of S2 and S3 does not affect S1, as S1 is independent of the other two. This does not mean that S1 cannot be rejected. Particularly for a wide sector of traditional symbolic cognitive sciences, S1 is considered false. I believe that S1 is a defensible assumption, though defending it carries the responsibility of accounting for the semantic referential relations between linguistic expressions and their referents. It is not the aim of this article to offer an explanatory alternative for phenomena such as reference relations, linguistic meaning, or human symbolic behavior. Nevertheless, the discussion presented here shows that behavior analysis has, in recent decades, focused its efforts on an explanatory paradigm that is inadequate for the very phenomena it seeks to explain. This highlights the existing shortcomings of this approach in providing a compelling account of issues related to complex human symbolic behavior

To conclude, I would like to say a few words about RFT. The arguments presented against S2 and S3 also affect the explanation of reference within RFT (e.g., Hayes & Grundt, 1997). These arguments become particularly problematic if it is maintained that the coordination frame is the most fundamental and serves as the foundation for the other relational frames (e.g., Hayes, Fox et al., 2021; Hughes & Barnes-Holmes, 2016a, 2016b). Now, if it is acknowledged that semantic word-referent relations cannot be reduced to a relation of sameness, RFT appears to be left with two alternatives: either reject S1 and consider that the referential relation is not the foundation of symbolic human behavior, or consider that such a relation can be explained by some other relational frame. However, both alternatives are problematic for RFT. The first because rejecting S1 is costly due to its plausibility, and even if one insisted on this, it would still be necessary to account for both the referential relations and its connection to the rest of human symbolic behavior. The second because it would be necessary to identify which relational frame could explain reference and demonstrate that it does not fall victim to counterexamples similar to those presented here. Another alternative is to postulate a relational frame specific to the referential relation. However, this approach carries the risk of creating a circular explanation.

## Data Availability

No datasets were generated or analyzed during the current study.
